# Childhood Sexual Victimization, Pedophilic Interest, and Antisocial Orientation

**DOI:** 10.5964/sotrap.5425

**Published:** 2022-04-11

**Authors:** Anna T. Pham, Kevin L. Nunes, Sacha Maimone, Sandy Jung

**Affiliations:** 1 Department of Psychology, Carleton University, Ottawa, Ontario, Canada; 2 Department of Psychology, MacEwan University, Edmonton, Alberta, Canada; University of Saskatchewan, Saskatoon, SK, Canada

**Keywords:** childhood sexual victimization, childhood abuse, sex offender against children, child pornography, risk status, psychopathy

## Abstract

According to the sexually abused-abuser hypothesis, childhood sexual victimization (CSV) among males increases the likelihood of later sexual offending against children. Why CSV is related to sexual offending against children, however, has yet to be determined. To explore mechanisms that may link CSV to sexual offending, we tested the relationship between CSV and the two main risk domains: pedophilic interest and antisocial orientation. In four studies, men convicted of sexual offences against children under 15 who reported experiencing CSV were generally more sexually interested in children—especially male children—and were more antisocial than those who did not report experiencing CSV. A meta-analysis of the results across our four studies showed that CSV was moderately associated with greater antisociality, but less so with pedophilic interests. Future research should test the extent to which pedophilic interest and antisocial orientation explain the relationship between CSV and sexual offending against children among convicted sexual offenders.

## Abstract (plain language summary)

**Background:**

Many theories suggest that childhood sexual victimization (CSV) plays an important role in later sexual offending against children, but how? Perhaps CSV leads to sexual interest in children and antisocial tendencies which leads to sexual offending against children for some people.

**Why was this study done?:**

The present paper further examines the relationship between CSV and indicators of pedophilic interest and antisocial tendencies in a series of studies.

**What did the researchers do and find?:**

We conducted statistical analyses on four datasets/samples that consisted of men convicted of sexual offences against children. Participants who reported experiencing CSV were generally more sexually interested in children—especially male children—and were more antisocial than those who did not report experiencing CSV. We averaged the results across the studies, and found that experiencing CSV was slightly related with more sexual interest in children, and was significantly related with more antisociality.

**What do these findings mean?:**

The findings from our paper and past research are consistent with (though certainly do not confirm) the hypothesis that CSV can lead to greater pedophilic interest and antisociality, which would increase the likelihood of sexual offending against children.

## Highlights

- Childhood sexual victimization (CSV) was generally related to offending against more children.
- There were mixed findings on the relationship between CSV and viewing child pornography.
- CSV was related to patterns of antisociality (more violence, higher risk to reoffend).
- CSV was related to more impulsivity/antisocial lifestyle.
- CSV was related to fewer interpersonal/affective psychopathy traits.

## General Introduction

Many theoretical models contend that childhood sexual victimization (CSV) plays an important role in later sexual offending against children (e.g., Burton, 2003; Johnson & Knight, 2000; Kobayashi et al., 1995; Marshall & Barbaree, 1990; Marshall & Marshall, 2000; Seto, 2018; Ward & Siegert, 2002). For example, the sexually abused-sexual abuser hypothesis posits that males who are sexually abused as children are at greater risk of sexually abusing children later in life (Garland & Dougher, 1990; Glasser et al., 2001). Consistent with this hypothesis, meta-analyses have reported higher prevalence rates of CSV in individuals convicted of sexual offences compared to those convicted of non-sexual offences (Jespersen et al., 2009; Seto & Lalumière, 2010; Whitaker et al., 2008). Further, those convicted of sexual offences against children, in particular, have higher rates of CSV than those convicted of sexual offences against adults, suggesting a specific link between CSV and sexual offending against children. Due to the methodological challenges of this line of research, the available evidence falls short of demonstrating a causal effect of CSV on sexual offending against children. But if there is in fact a causal effect as many have hypothesized, what would explain this effect? One suggested mechanism is that pedophilic interest and antisocial orientation mediate the relationship between CSV and sexual offending against children, as these are the two strongest risk factors for sexual offending (Hanson & Morton-Bourgon, 2005; McPhail et al., 2019; Seto, 2018).

Researchers have hypothesized that CSV may lead to the development of pedophilic interest for some victims, which may in turn lead to sexual offending against children (e.g., Burton, 2003; Freund & Kuban, 1994; Freund et al., 1990; Seto, 2018). Pedophilic interest would be reflected by greater sexual arousal to prepubescent children; sexual offending against prepubescent children, male children, and a greater number of children; and viewing child pornography (for a review, see Seto, 2018). The Screening Scale for Pedophilic Interests (SSPI; Seto & Lalumière, 2001) combines four offence history indicators of pedophilic interest (any male victims, more than one child victim, any victims under 12, and any extrafamilial child victims); higher scores on the SSPI are associated with greater sexual arousal to children (Seto & Lalumière, 2001). Studies have shown that CSV is related to these indicators of pedophilic interest. For example, CSV has been found to be associated with greater phallometric arousal to prepubescent children, greater likelihood of a psychiatric diagnosis of pedophilia, sexual offences against younger child victims, sexual offences against male children, child pornography offences, and higher scores on the SSPI (Craissati et al., 2002; Felson & Lane, 2009; Freund & Kuban, 1994; Freund et al., 1990; Nunes, Hermann, Malcom, & Lavoie, 2013; Ogloff et al., 2012; Seto & Lalumière, 2001; Widom, 2015; see Seto, 2018 for review).

Furthermore, theorists have speculated that CSV may cause desensitization to painful or anxiety-provoking experiences, callousness, empathy deficits, and disregard for the needs of others (Marshall & Barbaree, 1990). Thus, antisocial traits have been proposed as a mechanism mediating the relationship between CSV and later sexual offending against children (e.g., Christopher et al., 2007). Empirical studies have found that CSV is associated with greater antisociality as measured by a variety of indicators, such as risk of general recidivism, psychopathy, total number of offences, total number of non-sexual violent offences, and breaches of conditional release (e.g., bail, probation, parole; Craissati et al., 2002; Graham et al., 2012; McGrath et al., 2011; Ogloff et al., 2012; Spano et al., 2010).

However, with respect to psychopathy, CSV appears to be more related to generic antisocial and unstable lifestyle traits, rather than to the interpersonal and affective traits. In a sample of people convicted of violent offences, Schimmenti et al. (2015) found that CSV was significantly associated with higher scores on the lifestyle/antisocial behaviors items (Factor 2) of the Psychopathy Checklist-Revised (PCL-R; Hare, 2003), but CSV was not significantly associated with total scores on the PCL-R, suggesting that CSV may not be linked to psychopathy as a whole. Instead, CSV may be associated with only some characteristics of psychopathy, namely, unstable lifestyle/antisocial behaviors. Notwithstanding, CSV seems to be linked to indicators of antisocial orientation, as well as indicators of pedophilic interest.

The present paper further examines the association between CSV and the two strongest predictors of sexual offending against children: pedophilic interest and antisocial orientation. We conducted secondary analyses on four datasets that were originally gathered for larger projects. Though our datasets did not permit tests of whether pedophilic interest and antisocial orientation mediate the relationship between CSV and sexual offending against children, we were able to test the first step of this hypothesized mediation; that is, the relationship of CSV with pedophilic interest and antisocial orientation. Based on the reviewed literature, we hypothesized that men who experienced CSV would have higher SSPI scores, more sexual offences against children and specifically male children, and view more child pornography. With regard to antisociality, we hypothesized that those who experienced CSV would score higher on actuarial tools assessing risk of general violent recidivism, score higher on unstable lifestyle/antisocial behavior traits (i.e., according to self-report psychopathy scales and proxy scales assessing Factor 2 psychopathy traits), have more total and violent convictions, violate more release conditions, and be younger at their first conviction.

## Study 1

In this study, we examined the association of CSV with indicators of pedophilia (SSPI, sexual offending against prepubescent children and male children; and viewing child pornography) and indicators of antisocial orientation (risk assessment instrument scores, Factor 2 psychopathy traits, number of total and violent convictions, conditional release violations, and age at first conviction).

### Method

#### Participants

The data examined in Study 1 were drawn from a dataset originally collected for a larger project. The larger dataset has been previously used for other manuscripts (e.g., Jung et al., 2014; Jung et al., 2013; Jung et al., 2017); however, the research questions of interest in the current manuscript were not addressed in these previous studies. In Study 1, the sample consisted of 177 adult males convicted of at least one sexual offence against children 13 years or younger and whose files included information on their childhood experiences. These files were coded from a forensic psychiatric outpatient clinic in Alberta, Canada, and were referred for an assessment between 2009 and 2011. The average age of the sample was 37.50 years (*SD* = 13.50). The average level of education within the final sample was Grade 11.18 (*SD* = 2.34). Concerning marital status, over a third of the sample were single (*n* = 65; 36.7%) and almost a third were married (*n* = 53; 29.9%). Two-thirds had participated in prior treatment (*n* = 110; 62.1%), though completion rates were unknown.

#### Measures

##### Childhood Sexual Victimization (CSV)

Based on self-reported information documented in the file information (in the form of interview notes and written reports), individuals were classified as having experienced childhood sexual victimization if they were sexually abused and/or were exposed to sexual stimuli before the age of 13. Seventy-two (40.7%) men were classified as having experienced CSV prior to age 13 and 105 (59.3%) as not having experienced CSV.

##### Screening Scale for Pedophilic Interests (SSPI)

The SSPI (Seto & Lalumière, 2001) was designed to measure sexual interest in children among those who have sexually offended against children. Four items are scored from information commonly found in official records: (1) any male victims, (2) more than one child victim, (3) any victims under 12, and (4) any extrafamilial child victims. Scores can range from 0 to 5, with higher scores reflecting greater sexual interest in children. In terms of construct validity of the SSPI, it is correlated with phallometrically assessed sexual attraction to children (*r* = .23 and *r* = .27, Canales et al., 2009; *r* = .28, Seto et al., 2004; *r* = .34, Seto & Lalumière, 2001; *r* = .46, Seto et al., 2003).

##### Violence Risk Appraisal Guide (VRAG)

The VRAG (Harris et al., 1993) is an actuarial rating scale developed to assess risk for violent recidivism among adults convicted of violent offences. The scale consists of 12 items summed to form a total score that ranges from -26 to 38, with higher scores indicating a greater risk of violent reoffending. Loza and Dhaliwal (1997) found the internal consistency of the VRAG to be .72 among individuals convicted of offences who did not have a mental disorder. A meta-analysis demonstrated that the VRAG was a good predictor of violent (including sexual) recidivism (Hanson & Morton-Bourgon, 2009).

##### Child and Adolescent Taxon Scale (CATS)

The CATS (Harris et al., 1994) is an actuarial rating scale developed from variables related to childhood and adolescent antisocial and psychopathic characteristics. Consequently, it has been used as an alternative index of psychopathy. The CATS was developed as a proxy measure for inclusion in the VRAG and Sex Offender Risk Appraisal Guide (SORAG) in the absence of a PCL-R score (Quinsey et al., 2006). Harris et al. (1994) established that eight childhood variables could identify members of an antisocial/psychopathic class (e.g., teen alcohol abuse, childhood aggression, suspended or expelled from school). The eight items are scored and summed to form a total score that can range from 0 to 8. Higher CATS scores indicate more antisocial and psychopathic characteristics. The CATS has been found to have good internal consistency (α = .71) and excellent inter-rater reliability (*r* > .90; Glover et al., 2002; Skilling et al., 2002). The measure has demonstrated large correlations with PCL-R Factor 2 scores (*r* = .61 to .64; Glover et al., 2002; Skilling et al., 2002), but is less correlated with PCL-R Factor 1 scores (*r* = .08 to .35; Glover et al., 2002; Skilling et al., 2002).

##### Static-2002: General Criminality Subscale

The Static-2002 (Hanson & Thornton, 2003) contains 14 items grouped into five content areas, one of which is the General Criminality content area. For the purposes of this study, only the General Criminality raw score was used as one of the indicators of antisocial orientation. The General Criminality raw score is obtained by summing items 10 to 14 (“Any prior involvement with the Criminal Justice System,” “Prior sentencing occasions for anything,” “Any community supervision violation,” “Years free prior to index sex offence,” and “Any prior non-sexual violence sentencing occasion”) of the Static-2002. The raw score can range from 0-6, where higher scores indicate greater general criminality. Babchishin et al. (2016) found that the General Criminality subscale predicted nonsexual violent, any violent, and general recidivism significantly better than the Static-99R and Static-2002R (Hanson & Thornton, 2003; Helmus et al., 2012).

#### Statistical Analyses

Cohen’s *d* effect sizes (Cohen, 1992) were calculated to investigate the extent to which men who had versus those who had not experienced CSV differed on indicators of pedophilic interest and antisocial orientation. Effect sizes are statistically significant (α = .05) when the corresponding 95% confidence interval does not include zero. Cohen’s *d* effect sizes were calculated such that a positive value indicates that the *CSV* group was higher than the *no CSV* group on a given variable. While standards for interpreting the magnitude of effect sizes would ideally be tailored to specific research areas, the general convention is that a Cohen’s *d* of 0.20 is considered small, 0.50 is considered moderate, and 0.80 is considered large (Cohen, 1992).

#### Procedure

The present research was approved by an institutional research ethics board and also a separate ethics board designated to review research involving the use and disclosure of health information. Three undergraduate- and one graduate-level research assistants received a full day of training on the variables and were examined on three cases to ensure that they coded the variables reliably. All variables examined in the current study were coded by research assistants retrospectively from official records in participants’ case files, which contained interview notes (self-reported information), formal assessment reports by the assessor at the clinic, official criminal records and police documentations provided with the referral, any prior collateral reports from correctional institutions and/or probation, demographic information, and some description of victim information. Most variables (e.g., CSV, pornography use) were coded based on self-report documented in the individuals’ files, unless collateral documentation provided details (e.g., police reports, correctional reports, previous assessments). Of note, due to some missing information, sample sizes may vary depending on the variables included in the analyses.

### Results

Table 1 displays means, standard deviations, and Cohen’s *d* effect sizes for comparisons between men convicted of sexual offences against children who had versus those who had not experienced CSV before the age of 13 on indicators of pedophilic interest. Compared to those with no CSV, those who had experienced CSV had higher SSPI scores (*d* = 0.19). Further, participants who had experienced CSV offended against more male victims under 13 (*d* = 0.26). Overall, those who had been sexually victimized as children also offended against more victims under 13 (*d* = 0.17). However, CSV was not associated with more child pornography use (*d* = 0.04). Most associations between CSV and pedophilic interest were in the expected direction, and generally consistent with past research (e.g., Freund et al., 1990; Freund & Kuban, 1994; Nunes, Hermann, Malcom, & Lavoie, 2013).

**Table 1:** Comparison of Sexual Offenders Against Children by Childhood Sexual Victimization (CSV) on Indicators of Pedophilic Interest in Study 1

Variable	CSV < 13 years old		Cohen’s *d* [95% CI]
	Yes *M* (*SD*)	No *M* (*SD*)	
SSPI	2.36 (1.51) *n* = 72	2.10 (1.29) *n* = 105	0.19 [-0.12, 0.49]
# of Victims Under 13^a^	0.31 (0.17) *n* = 69	0.28 (0.15) *n* = 101	0.17 [-0.14, 0.48]
# of Male Victims Under 13	0.23 (0.52) *n* = 69	0.12 (0.36) *n* = 100	0.26 [-0.05, 0.57]
Child Pornography Use	0.20 (0.41) *n* = 40	0.19 (0.39) *n* = 70	0.04 [-0.35, 0.42]

*Note.* All variables were coded from official documentation.

^a^Variable was log transformed.

With regard to the relationship between CSV and indicators of antisocial orientation (see Table 2), compared to those with no CSV, those who had experienced CSV had a higher risk of reoffending violently (i.e., VRAG scores; *d* = 0.87), higher General Criminality subscore (*d* = 0.21), and possessed more psychopathy traits (i.e., CATS scores; *d* = 0.74). Those who had experienced CSV also had more total past convictions (*d* = 0.35), particularly prior violent convictions (*d* = 0.40). Participants also violated more release conditions (*d* = 0.28) if they had experienced CSV. Interestingly, those who experienced CSV were also younger at their first conviction (*d* = -0.33). All results showed the expected association between CSV and indicators of antisocial orientation, and were consistent with past research.

**Table 2:** Comparison of Sexual Offenders Against Children by Childhood Sexual Victimization (CSV) on Indicators of Antisocial Orientation in Study 1

Variable	CSV < 13 years old		Cohen’s *d* [95% CI]
	Yes *M* (*SD*)	No *M* (*SD*)	
VRAG	4.19 (10.39) *n* = 31	-4.41 (9.48) *n* = 46	0.87* [0.04, 1.35]
CATS	3.55 (2.73) *n* = 33	1.84 (2.04) *n* = 56	0.74* [0.29, 1.18]
Static-2002: General Criminality Subscore	1.81 (1.89) *n* = 74	1.45 (1.61) *n* = 107	0.21 [-0.09, 0.51]
Total Past Convictions	6.03 (8.66) *n* = 73	3.34 (6.72) *n* = 105	0.35* [0.05, 0.66]
Prior Violent Convictions	1.21 (1.77) *n* = 72	0.60 (1.29) *n* = 105	0.40* [0.10, 0.71]
Conditional Release Violations	0.32 (0.47) *n* = 68	0.20 (0.40) *n* = 99	0.28 [-0.03, 0.59]
Age at First Conviction	26.69 (14.70) *n* = 68	31.71 (15.53) *n* = 90	-0.33* [-0.65, -0.01]

*Note.* All variables were coded from official documentation.

*p < .05.

## Study 2

In this study, we attempted to replicate results found in Study 1.

### Method

#### Participants

The data examined in this study were drawn from a dataset originally collected for a larger project. The larger dataset has been previously used in a thesis, conference presentations, and manuscript (e.g., Hermann et al., 2013; Kostiuk, 2012; Nunes, Hermann, Maimone, et al., 2013; Pettersen et al., 2013; Pettersen et al., 2018); however, the research questions of interest in the current manuscript were not addressed in these previous studies. In Study 2, participants were adult males convicted of sexual offences against children who were serving provincial and federal sentences within medium- and maximum- security prisons, as well as from community supervision and treatment services in Western Canada. The sample consisted of 28 adult males who offended against children 15 years or younger. The average age of the sample was 35.32 years (*SD* = 12.62). The majority of participants were White (*n* = 23; 82.1%) and the rest were Indigenous/Metis (*n* = 5; 17.9%). Over half were single (*n* = 17; 60.7%), and the rest were either married/common-law (*n* = 7; 25.0%) or separated/divorced (*n* = 4; 14.3%). Almost half of the sample (*n* = 12; 42.9%) did not complete Grade 12.

#### Measures

The SSPI, VRAG, and CATS (see Study 1 Measures) were also used in Study 2.

##### Childhood Sexual Victimization (CSV)

As part of a comprehensive interview, participants were asked if they had experienced sexual abuse before the age of 12. Thirteen participants (out of 28) reported experiencing sexual abuse before the age of 12 and 15 participants reported no sexual abuse before the age of 12.

##### Self-Report Psychopathy scale-III (SRP-III)

The SRP-III (Williams et al., 2007) is a 64-item, self-report measure designed to assess psychopathy, as well as four facets of psychopathy, which are Interpersonal Manipulation (IPM), Callous Affect (CA), Erratic Lifestyle (ELS), and Criminal Tendencies (CT). Items are scored on a 5-point Likert scale (*disagree strongly* to *agree strongly*) and are then summed to provide subscale or total scores, where higher scores indicate more psychopathic characteristics. The SRP-III subscales have been shown to be reliable (e.g., IPM, α = .82; CA, α = .79; ELS, α = .81; CT, α = .68; Seibert et al., 2011). The SRP-III total scores were also positively and significantly correlated with the PCL-R total scores (*r* = .44, *p* < .001; Sandvik et al., 2012). In addition, the ELS and CT subscales had the highest correlations to the PCL-R Facet 3 (i.e., lifestyle instability; *r* = .52, *p* < .001) and Facet 4 (i.e., antisocial behavior; *r* = .66, *p* < .001), respectively. However, the SRP-III total and subscales were not related to the PCL-R Facet 1 or Facet 2 (Sandvik et al., 2012).

##### Self-Report Questionnaire

A self-report questionnaire was given to participants to complete under conditions of confidentiality. The questionnaire was developed for the original project and included questions on demographic and criminal history information. Some self-reported questions from the questionnaire included in this study were “Have you ever been charged with a sex offence against someone under the age of 12?” (0 = *Never* to 9 = *9 times or more*), “How many boys under the age of 12 have you had sexual contact with when you were at least 5 years older than them?” (0 = *Never* to 9 = *9 or more*), and “Have you ever looked at child pornography?” (0 = *Never* to 9 = *9 times or more*).

#### Statistical Analyses

As in Study 1, Cohen’s *d* effect sizes were calculated to investigate the strength and direction of the relationships of CSV with indicators of pedophilic interest and antisocial orientation.

#### Procedure

Data for this study were collected as part of a larger project. The study was approved by the university research ethics board and the correctional agency. Similar to Study 1, files were coded retrospectively and no additional measures were administered for the purpose of this study. At the time of data collection, participants completed a questionnaire concerning demographic and offence history information, after consenting to participate. Therefore, available variables consisted of information collected from both official records and self-report.

### Results

Table 3 displays means, standard deviations, and Cohen’s *d* effect sizes for comparisons between men convicted of sexual offences who had experienced CSV before the age of 12 and those who had not on indicators of pedophilic interest.

**Table 3:** Comparison of Sexual Offenders Against Children by Childhood Sexual Victimization (CSV) on Indicators of Pedophilic Interest in Study 2

Variable	CSV < 12 years old		Cohen’s *d* [95% CI]
	Yes *M* (*SD*)	No *M* (*SD*)	
Official Documentation			
SSPI	2.85 (1.52) *n* = 13	2.40 (1.30) *n* = 15	0.32 [-0.43, 1.07]
Self-Report			
# of Charges Against Victims Under 12^a^	0.30 (0.35) *n* = 12	0.19 (0.31) *n* = 15	0.34 [-0.43, 1.10]
# of Male Victims Under 12^b, c^	0.13 (0.24) *n* = 10	0.15 (0.33) *n* = 10	-0.06 [-0.94, 0.82]
Child Pornography Use	4.08 (4.33) *n* = 13	3.93 (4.37) *n* = 15	0.03 [-0.71, 0.78]

^a^Only includes victims from reported offences. ^b^Includes all victims (i.e., from reported and unreported offences). ^c^Variable was log transformed.

 As in Study 1, those who reported experiencing CSV had higher SSPI scores (*d* = 0.32) and had more charges against victims under 12 (*d* = 0.34) compared to the no CSV group. However, there was no difference in number of male victims under 12 (*d* = -0.06). As in Study 1, there was no difference between groups in child pornography use (*d* = 0.03). In this study, only the association between CSV, SSPI scores, and number of charges against victims under 12 were in the expected direction and consistent with past research.

With regard to the relationship between CSV and indicators of antisocial orientation (see Table 4), compared to those who reported no CSV, those who reported experiencing CSV had a higher risk of reoffending violently (i.e., VRAG scores; *d* = 0.25) and had more psychopathy traits according to their CATS scores (*d* = 0.44).

As for self-reported psychopathy (i.e., SRP-III), participants who reported experiencing CSV had higher criminal tendencies (*d* = 0.34), which is a Factor 2 (i.e., unstable lifestyle/antisocial behavior) subscale. However, there was virtually no difference between groups in terms of erratic lifestyle (*d* = 0.09), which is also a Factor 2 subscale. In terms of Factor 1 (i.e., interpersonal and affective traits) subscales, those who experienced CSV scored lower than those who did not experience CSV (i.e., IPM, *d* = -0.15; CA, *d* = -0.28). Generally, the direction of these relationships was expected given that past research has found that CSV was more associated with impulsive-irresponsible lifestyle and antisocial conduct, rather than interpersonal and affective traits of psychopathy (e.g., Schimmenti et al., 2015). Nevertheless, given these inconsistent directions of Cohen’s *d* effect sizes, it is not surprising that there was no difference in total psychopathy score (*d* = 0.04). With regard to criminal history information, those who reported CSV had more total past convictions (*d* = 0.58), prior violent convictions (*d* = 0.73), and conditional release violations (*d* = 0.41). All results were in the expected direction between CSV and indicators of antisocial orientation, and were consistent with Study 1 and past research.

**Table 4:** Comparison of Sexual Offenders Against Children by Childhood Sexual Victimization (CSV) on Indicators of Antisocial Orientation in Study 2

Variable	CSV < 12 years old		Cohen’s *d* [95% CI]
	Yes *M* (*SD*) *n* = 13	No *M* (*SD*) *n* = 15	
Official Documentation			
VRAG	3.77 (7.65)	1.40 (10.78)	0.25 [-0.50, 1.00]
CATS	0.77 (2.45)	-0.33 (2.58)	0.44 [-0.31, 1.19]
Total Past Convictions	0.61 (0.55)	0.31 (0.49)	0.58 [-0.18, 1.33]
Self-Report			
Prior Violent Convictions	2.00 (3.24)	0.33 (0.82)	0.73 [-0.04, 1.50]
Conditional Release Violations	1.00 (2.20)	0.33 (0.82)	0.41 [-0.34, 1.16]
SRP: Interpersonal Manipulation	35.08 (7.16)	36.20 (7.76)	-0.15 [-0.89, 0.59]
SRP: Callous Affect	35.08 (8.17)	37.33 (7.74)	-0.28 [-1.03, 0.46]
SRP: Erratic Lifestyle	46.00 (7.21)	45.33 (8.13)	0.09 [-0.66, 0.83]
SRP: Criminal Tendencies	41.62 (11.35)	37.93 (10.35)	0.34 [-0.41, 1.09]
SRP: Total	157.77 (23.75)	156.80 (27.86)	0.04 [-0.71, 0.78]

## Study 3

In this study, we attempted to replicate results, as well as clarify inconsistent findings, found in the previous studies.

### Method

#### Participants

The data examined in this study were drawn from a dataset originally collected for a larger project. The larger dataset has been previously used in manuscripts and a conference presentation (e.g., Babchishin et al., 2015); however, the research questions of interest in the current manuscript were not addressed in these previous studies. In Study 3, participants were recruited from a maximum-security correctional institution in Ontario, Canada. The sample consisted of 27 adult males who offended against children 15 years or younger. The average age of the sample was 35.15 years (*SD* = 8.82). One-third of the sample (*n* = 9; 33.3%) completed college/university and 29.6% (*n* = 8) did not complete high school. Almost three-quarters (*n* = 20; 74.1%) of the sample were White. The rest were Asian (*n* = 1; 3.7%), Indigenous (*n* = 1; 3.7%), Metis (*n* = 1; 3.7%), African Canadian (*n* = 1; 3.7%), and other (*n* = 2; 7.4%).

#### Measures

The SSPI and the self-report questionnaire from Study 2 were also used in Study 3.

##### Childhood Sexual Victimization (CSV)

As part of a larger questionnaire, participants were asked to self-report the number of times they had been sexually abused before the age of 12. Participants responded on a 10-point scale ranging from 0 (*never*) to 9 (*9 times or more*). Participants who had reported one or more such experiences were classified as having experienced CSV before the age of 12 (*n* = 13), and those who reported no such experiences were classified as having not experienced CSV (*n* = 14).

##### Statistical Information on Recidivism—Revised 1 (SIR-R1)

The SIR-R1 (Nafekh & Motiuk, 2002) assesses risk of general recidivism within three years of release, by combining 15 items in a scoring system. Simple summation of the SIR-R1 item scores yields a total score ranging from -30 to +27, with higher scores indicating a lower risk of general reoffending. A recent meta-analysis (Hanson & Morton-Bourgon, 2009) demonstrated that the SIR-R1 was a good predictor of violent (including sexual) recidivism. SIR-R1 scores were extracted from participants’ files when available, and were not scored by researchers. Note that SIR-R1 scores were not available for Indigenous participants.

#### Statistical Analyses

Again, Cohen’s *d* effect sizes were calculated to investigate the strength and direction of the relationships of CSV with indicators of pedophilic interest and antisocial orientation.

#### Procedure

Procedure for this study was identical to the procedure in Study 2. The study was approved by the university research ethics board and the correctional agencies.

### Results

Table 5 displays means, standard deviations, and Cohen’s *d* effect sizes for comparisons between men convicted of sexual offences against children who had experienced CSV before the age of 12 and those who had not on indicators of pedophilic interest. Similar to Studies 1 and 2, those who reported experiencing CSV had higher SSPI scores—though the effect size was quite small (*d* = 0.15)—and offended against more male victims under 12 (*d* = 0.52), compared to those who reported no CSV. However, those who reported CSV had fewer charges against victims under the age of 12 (*d* = -0.35). Participants who experienced CSV in this study also used more child pornography (*d* = 0.20), which is inconsistent with what we found in Studies 1 and 2, but is consistent with the broader literature. Additionally, we found that age of the youngest victim was younger for those who reported no CSV (*d* = 0.42), compared to those who reported CSV, which was unexpected and inconsistent with past research. In this study, only SSPI scores, number of male victims, and child pornography use were in the expected direction and consistent with past research, even though most of these effect sizes were small.

**Table 5:** Comparison of Sexual Offenders Against Children by Childhood Sexual Victimization (CSV) on Indicators of Pedophilic Interest in Study 3

Variable	CSV < 12 years old		Cohen’s *d* [95% CI]
	Yes *M* (*SD*) *n* = 13	No *M* (*SD*) *n* = 14	
Official Documentation			
SSPI	2.54 (1.39)	2.36 (1.08)	0.15 [-0.61, 0.90]
Age of Youngest Victim	7.31 (2.36)	6.21 (2.78)	0.42 [-0.34, 1.19]
Self-Report			
# of Charges Against Victims Under 12^a^	0.62 (0.77)	0.93 (1.00)	-0.35 [-1.11, 0.41]
# of Male Victims Under 12^b^	0.31 (0.85)	0.00 (0.00)	0.52 [-0.25, 1.29]
Child Pornography Use	2.92 (4.05)	2.14 (3.76)	0.20 [-0.56, 0.96]

^a^Only includes victims from reported offences. ^b^Includes all victims (i.e., from reported and unreported offences).

With regard to the relationship between CSV and indicators of antisocial orientation (see Table 6), men who reported experiencing CSV had lower SIR-R1 scores (*d* = -0.79), indicating a higher risk of general recidivism for those who experienced CSV. Unlike our previous studies, those who experienced CSV had fewer conditional release violations (*d* = -0.18) and did not differ on prior violent convictions (*d* = 0.04). In this study, only SIR-R1 was in the expected direction and consistent with past research.

**Table 6:** Comparison of Sexual Offenders Against Children by Childhood Sexual Victimization (CSV) on Indicators of Antisocial Orientation in Study 3

Variable	CSV < 12 years old		Cohen’s *d* [95% CI]
	Yes *M* (*SD*)	No *M* (*SD*)	
Official Documentation			
SIR-R1	9.83 (7.18) *n* = 12	15.69 (7.64) *n* = 14	-0.79 [-1.59, 0.01]
Self-Report			
Prior Violent Convictions^a^	0.14 (0.26) *n* = 13	0.13 (0.33) *n* = 14	0.04 [-0.71, 0.80]
Conditional Release Violations^a^	0.02 (0.08) *n* = 13	0.05 (0.19) *n* = 14	-0.18 [-0.94, 0.57]

*Note*. Lower SIR-R1 scores reflect higher risk of general recidivism.

^a^Variable was log transformed.

## Study 4

This study was conducted as another attempt to replicate or clarify results found in the previous studies.

### Method

#### Participants

The data examined in this study were drawn from a dataset originally collected for a larger project. The larger dataset has been previously used in a thesis, conference presentations, and manuscript (e.g., McPhail, 2010; McPhail et al., 2018; McPhail et al., 2010); however, the research questions of interest in the current manuscript were not addressed in these previous studies. In Study 4, participants were recruited from medium- and maximum-security forensic and correctional institutions in Ontario, Canada. The sample consisted of 30 adult males who offended against children 15 years or younger. Of the 30 men, 13 were missing information on childhood victimization and/or did not understand written English, and were thus excluded from further analyses. The final sample consisted of 17 adult males convicted of at least one sexual offence against a child. The average age of the sample was 43.23 years (*SD* = 10.30). Over one-third (*n* = 6; 35.3%) of the sample completed high school. Most were White (*n* = 11; 64.7%) and the rest were Indigenous (*n* = 1; 5.9%) or other ethnicities (*n* = 2; 11.8%). Note that three (17.6%) participants were missing ethnicity information.

#### Measures

The SSPI, SIR-R1, and Self-Report Questionnaire (see Study 1 and 3 Measures section) were also used in Study 4.

##### Childhood Sexual Victimization (CSV)

The measurement and operationalization for CSV in this study were the same as those in Study 3. Eight out of 17 participants reported experiencing CSV and nine reported never experiencing CSV.

#### Statistical Analyses

Again, Cohen’s *d* effect sizes were calculated to investigate the strength and direction of the relationships of CSV with indicators of pedophilic interest and antisocial orientation.

#### Procedure

Procedure for this study was identical to that in Study 2. The study was approved by our university research ethics board and the correctional/forensic agencies.

### Results

Table 7 displays means, standard deviations, and Cohen’s *d* effect sizes for comparisons between men convicted of sexual offences against children who had experienced CSV before the age of 12 and those who had not on indicators of pedophilic interest. Consistent with our previous studies, men who reported CSV had higher SSPI scores (*d* = 0.25) and had more male child victims (*d* = 1.14) compared to those who reported no CSV. Different from Studies 1 and 2, but consistent with Study 3 and the broader literature, those who reported CSV used more child pornography (*d* = 0.44). Unlike Study 3—but consistent with the other studies—those who reported CSV had more charges against victims under 12 (*d* = 1.89) and offended against younger victims (*d* = -1.66). All results on the indicators of pedophilic interest were in the expected direction and consistent with past research.

**Table 7:** Comparison of Sexual Offenders Against Children by Childhood Sexual Victimization (CSV) on Indicators of Pedophilic Interest in Study 4

Variable	CSV < 12 years old		Cohen’s *d* [95% CI]
	Yes *M* (*SD*)	No *M* (*SD*)	
Official Documentation			
SSPI	4.00 (1.51) *n* = 8	3.60 (1.67) *n* = 5	0.25 [-0.87, 1.38]
Age of Youngest Victim	6.25 (2.82) *n* = 8	10.78 (2.64) *n* = 9	-1.66* [-2.77, -0.56]
Self-Report			
# of Charges Against Victims Under 12^a^	0.48 (0.31) *n* = 8	0.04 (0.11) *n* = 8	1.89* [0.71, 3.07]
# of Male Victims Under 12	3.43 (3.87) *n* = 7	0.00 (0.00) *n* = 5	1.14 [-0.09, 2.38]
Child Pornography Use	3.00 (4.07) *n* = 8	1.44 (2.92) *n* = 9	0.44 [-0.52, 1.41]

^a^Variable was log transformed.

*p < .05.

With regard to the relationship between CSV and indicators of antisocial orientation (see Table 8), similar to Study 3, men who reported experiencing CSV had lower SIR-R1 scores (*d* = -0.70), indicating that they are at higher risk of general recidivism. As expected, CSV was associated with more prior violent convictions (*d* = 0.39). However, those who experienced CSV had fewer violations of conditional release, though this difference was small (*d* = -0.10). Most of the results on the indicators of antisocial orientation in this study were in the expected direction and consistent with past research.

**Table 8:** Comparison of Sexual Offenders Against Children by Childhood Sexual Victimization (CSV) on Indicators of Antisocial Orientation in Study 4

Variable	CSV < 12 years old		Cohen’s *d* [95% CI]
	Yes *M* (*SD*)	No *M* (*SD*)	
Official Documentation			
SIR-R1	2.50 (7.67) *n* = 8	8.17 (8.59) *n* = 6	-0.70 [-1.79, 0.39]
Self-Report			
Prior Violent Convictions	2.88 (3.76) *n* = 8	1.67 (2.29) *n* = 9	0.39 [-0.57, 1.36]
Conditional Release Violations^a^	0.10 (0.19) *n* = 8	0.12 (0.27) *n* = 9	-0.10 [-1.05, 0.86]

*Note*. Lower SIR-R1 scores reflect higher risk of general recidivism.

^a^Variable was log transformed.

## Meta-Analysis of Group Differences From Studies 1-4

Although results were generally consistent across Studies 1 to 4 for some variables, results for other variables seemed to vary and we often lacked adequate statistical power due to small sample sizes. Therefore, to synthesize findings, we conducted a fixed-effects meta-analysis of the observed group differences of CSV with indicators of pedophilic interest and antisocial orientation. We examined the combined and synthesized effects of the two broad domains (i.e., indicators of pedophilic interest and indicators of antisocial orientation) across all four studies.

### Method

#### Statistical Analyses

Within each study, we combined Cohen’s *d* effect sizes for all indicators of pedophilic interest, and similarly for all indicators of antisocial orientation. More specifically, all effect sizes included in the pedophilic interest meta-analysis were from all the comparisons reported in Tables 1, 3, 5, and 7. Likewise, all effect sizes included in the antisocial orientation meta-analysis were from all the comparisons reported in Tables 2, 4, 6, and 8. We subsequently synthesized the combined effects of indicators of pedophilic interest and indicators of antisocial orientation across all four studies using Comprehensive Meta-Analysis (Biostat, 2006). Note that we also revised the direction of the effect size of some variables when needed to keep the variables more consistent. For example, we revised the direction of the effect size for the age of youngest victim variable, because a negative effect size would indicate more pedophilic interest among the CSV group.

### Results

Overall, the combined effect sizes from all four studies demonstrated that experiencing CSV was associated with slightly more pedophilic interests than not experiencing CSV (fixed effects weighted average *d* = 0.22, 95% CI [-0.06, 0.50], *Q*[3] = 2.32, *I*^2^ = 0.00, *k* = 4), though this difference was not significant. Additionally, combined effect sizes showed that those who experienced CSV had significantly and moderately greater antisocial orientation (fixed effects weighted average *d* = 0.38, 95% CI [0.08, 0.68], *Q*[3] = 0.43, *I*^2^ = 0.00, *k* = 4). Generally, the results from our meta-analysis were in the expected direction and consistent with past research.

## Discussion

The first goal of this paper was to examine the relationship between CSV and indicators of pedophilic interest. Although the relationship between CSV and SSPI scores did not reach significance in any of the studies, our findings remain in the direction that is consistent with the literature across all four studies. Specifically, we found that those who had experienced CSV before the age of 12 or 13 were more pedophilic (as indicated by their SSPI scores), which is consistent with the literature (e.g., Nunes, Hermann, Malcom, & Lavoie, 2013; Seto, 2018; Seto & Lalumière, 2010). In addition, with the exception of Study 3, our results generally suggest that those who experienced CSV offended against, and had more charges against, more children (of either gender) under 12 or 13, though these relationships, again, did not reach significance except for Study 4. We also found that experiencing CSV was generally—albeit not significantly—related to offending against more male children specifically across three out of four samples of men convicted of sexual offences against children.

On the other hand, our findings were mixed with respect to the age of the youngest victim. For example, results from Study 4 indicated that men who experienced CSV offended against significantly younger child victims, whereas results from Study 3 indicated that men who experienced CSV offended against older victims, but this finding was not significant. Furthermore, even though Studies 3 and 4 demonstrated that men who experienced CSV were generally more likely to view child pornography, Studies 1 and 2 showed no difference between the two groups, which is inconsistent with past findings. However, it is important to note that these relationships were not significant. Not only is viewing child pornography a criminal offence, it is also a social taboo; thus, some participants may have been fearful to disclose this information.

These mixed findings could also reflect the differences in our samples and settings. For example, Studies 1 and 2 participants were recruited both from prisons and outpatient services in Western Canada, whereas participants in other samples were all incarcerated at the time of these studies. Therefore, it is possible that the former samples may have been less comfortable disclosing their offences against children and other victim information (as these are more socially unacceptable crimes) and may have withheld their offence information due to fear of potential legal repercussions (e.g., jail time), particularly those who were free in the community. Additionally, it is also possible that some discrepant findings across our studies resulted from the differing risk levels among our samples. For example, the average SIR-R1 scores in Studies 3 and 4 were 14.30 and 5.42, respectively. This substantial variance in level of general risk between the two studies could account for the fact that Study 3 results indicated a negative relationship between CSV and the number of charges for sexual offences against victims under 12, whereas Study 4 results indicated a significant and positive relationship between these two variables. In fact, Nunes, Hermann, Malcom, and Lavoie (2013) found that actuarial risk moderated the relationship between CSV and sexual recidivism, such that CSV predicted sexual recidivism but only for those in the higher risk category. However, the researchers did not examine whether actuarial risk moderated the relationship between CSV and indicators of pedophilic interest. Unfortunately, our small sample sizes did not lend enough statistical power to examine the effects of different risk levels of these relationships. Future research should replicate these findings with a larger sample size to be able to statistically control for risk level, and examine whether risk level could moderate the relationship between CSV and indicators of pedophilic interest.

The second goal of this paper was to examine the relationship between CSV and indicators of antisocial orientation. Consistent with past research (e.g., Craissati et al., 2002; Levenson & Socia, 2016; Ogloff et al., 2012; Widom, 1996), we found that men who had experienced CSV before the age of 12 or 13 tended to be higher risk to reoffend generally and violently, have more total past convictions and were younger in age at their first conviction. Although these relationships approached significance only in Study 1, results from the other studies were, nevertheless, in the expected direction. We also found that, with the exception of Study 3, those who experienced CSV had more prior violent convictions (this finding was significant for Study 1), which was consistent with past research (e.g., Ogloff et al., 2012). However, our findings were mixed with respect to conditional release violations, as Studies 1 and 2 showed that men who experienced CSV had more violations, whereas Studies 3 and 4 results were in the opposite direction. This was inconsistent with past research (e.g., Ogloff et al., 2012).

Interestingly, we found that CSV was significantly and positively associated with criminality/antisocial lifestyle, as assessed by the CATS in Studies 1 and 2. When psychopathy was assessed using the SRP in Study 2, findings indicated that CSV was related to criminality/antisocial lifestyle, but negatively associated with interpersonal and affective psychopathy traits, though these results did not approach significance. These results are consistent with some past research (e.g., Graham et al., 2012; Schimmenti et al., 2015). However, a recent study by Grady et al. (2019) found that childhood abuse experiences among individuals who commit sexual offences were not significantly related to any of the PCL-R’s four facets (i.e., Interpersonal, Affective, Lifestyle, and Antisocial facets). Notably, Grady and colleagues did not distinguish between childhood sexual abuse and childhood physical abuse, which could explain the differing results. For example, childhood sexual abuse is generally more positively related to PCL-R total, and in particular, the Lifestyle and Antisocial facets, whereas childhood physical abuse is only marginally related to the psychopathy total score (e.g., Graham et al., 2012), suggesting that subtypes of childhood abuse experiences may be differentially related to psychopathic features. Therefore, combining childhood sexual and physical abuse experiences in Grady et al.’s (2019) study may have masked the specific relationship between CSV and certain features of psychopathy. Notwithstanding, our results are consistent with Schimmenti et al.’s (2015) finding that CSV was significantly associated with higher scores on PCL-R Factor 2, which reflects antisocial lifestyle features, but was not associated with scores on PCL-R Factor 1, which reflects interpersonal and affective traits. Taken together, the available evidence suggests that experiencing CSV is associated with more antisocial features of psychopathy (Factor 2) and perhaps fewer interpersonal/affective features of psychopathy (Factor 1).

From a neurobiological perspective, CSV has been found to be associated with long-term dysfunction in emotional and behavioral outcomes (e.g., Anda et al., 2006; Luby et al., 2019; Perry, 2008), as higher brain regions responsible for thinking and emotion regulation develop early in childhood. Traumatic childhood experiences, especially sexual abuse in both the preschool (Fresno et al., 2014; Langevin et al., 2016) and school-aged (Gauthier-Duchesne et al., 2017; Hébert et al., 2016) period, may be related to alterations in the development of this region of the brain, potentially influencing dysfunctional brain patterns that may ultimately lead to psychopathology, which is often expressed as emotional or relational problems, such as antisocial traits and behaviors (Luby et al., 2019; Perry, 2008). Therefore, it is possible that CSV can lead to antisocial traits and behaviors through the altered development of brain functioning. In addition, CSV is associated with disrupted social functioning, a risk factor for developing antisocial behavior, while antisocial behavior can cause difficulty in relating to others (Molnar et al., 2001).

We conducted a meta-analysis of the observed group differences to quantitatively synthesize the findings across our four studies. As expected, we found that men who experienced CSV had more antisocial features than those who had not experienced CSV. Further, though the average difference in pedophilic interests did not reach statistical significance, it was in the expected direction; that is, men convicted of sexual offences against children who experienced CSV had more pedophilic interests than those who had not experienced such abuse. Overall, our meta-analytic results were consistent with the broader literature.

Some limitations in our studies should be noted. First, as mentioned above, most of our sample sizes were quite small, which, not only affects our ability to generalize our findings, but also limits our statistical power to conduct more complex and advanced statistical analyses. Certainly, relationships between psychologically meaningful constructs are often influenced by additional/confounding variables; thus, the relationship between experiencing CSV and endorsing indicators of pedophilic interest and antisocial orientation could be further probed for the influence of additional variables, such as risk level, to more fully understand the path through which CSV may lead to pedophilic interest and antisocial orientation. However, we did not have enough power to conduct more sophisticated analyses, such as moderated regression; therefore, future research should further investigate how CSV may be related to pedophilic interest and antisocial orientation, and whether other variables may have an impact on this relationship.

Second, most variables included in Studies 2-4 were self-report (e.g., number of charges against victims and males under the age of 12, child pornography use, prior violent convictions, conditional release violations, etc.) and some would argue that the information may not be as reliable as information from official documentation. Relatedly, many of these self-report variables were based on single items with untested validity. Indeed, some researchers have raised concerns about the accuracy of self-report data (e.g., Lalumière et al., 2005), as it is possible participants may not be able to accurately recall or otherwise choose to misrepresent their sexuality and past offences, particularly if such offences are socially seen as heinous. Although this is a possibility, there is considerable evidence for the reliability and validity of self-reported information to assess engagement in delinquency and crime, including sexual aggression (see Piquero et al., 2014, and Thornberry & Krohn, 2000, for overviews; Pham et al., 2021; Weinrott & Saylor, 1991). Relatedly, we did not assess social desirability. However, prior studies have shown that the extent of socially desirable responses as typically measured is smaller than previously assumed and its impact on self-reports is low (e.g., Kroner et al., 2007), suggesting these results are likely still reliable despite the lack of controlling for social desirability. Moreover, the larger datasets did not contain interrater reliability information for the coding of our file-coded variables of interest; therefore, we were unable to examine raters’ reliability. As a result, this could lead to errors in coding and ultimately, render our results inaccurate or unreliable. However, it is important to note that our overall results are generally consistent with previous studies, suggesting the coding may be reliable. Nevertheless, future research should be more diligent in collecting interrater information to ensure fidelity of coding, as well as accuracy in reported results.

A third limitation is that the definition of CSV in Study 1 includes exposure to sexual stimuli. However, research shows that children under the age of 13 may be watching or seeking out pornography of their own accord (Wright, 2014; Ybarra & Mitchell, 2005), which does not classify as sexual abuse. Therefore, our definition of CSV may be overly inclusive, which would bias our results. Nevertheless, the majority (87%) of youth who report looking for sexual images online are 14 years of age or older (Ybarra & Mitchell, 2005); thus, it is possible that our definition captured those whose experiences would classify as abuse. In addition, despite the variation in definition of abuse among our studies, the findings were relatively consistent across studies, suggesting that the results remain valid.

Finally, all of our studies were cross-sectional; therefore, we were unable to determine a causal relationship, or even the potential direction of influence, between variables. Using non-experimental longitudinal designs and statistically controlling for potential confounds would provide more convincing circumstantial evidence regarding the potential causal effects of CSV. Additionally, though the purpose of our meta-analysis was to synthesize findings across our four studies, the number of studies was rather small (*k* = 4) for meta-analyses. Moreover, our studies did not fully match on operationalization of CSV (e.g., CSV before age 12 versus 13) and we had varying participant demographics; therefore, the samples may not have been fully comparable. Nevertheless, there was homogeneity among the study effect sizes that contributed to the overall mean effect sizes (*Q* values were non-significant and *I*^2^ values were zero).

Notwithstanding these limitations, the results from the current paper and past research generally suggest that CSV may be linked to greater sexual interest in children and greater antisociality among convicted sexual offenders, which are both important predictors of sexual offending against children. Future research should use more rigorous methodology to further examine the extent to which CSV impacts pedophilic interest, antisociality, and sexual offending, as well as whether pedophilic interest and antisociality mediate the relationship between CSV and sexual offending.

## Data Availability

The data for this research study will not be available to other researchers.
